# Stage-specific prevalence and progression of sarcopenia among aging hemodialysis patients: a multicenter cross-sectional study

**DOI:** 10.3389/fpubh.2025.1676139

**Published:** 2025-11-06

**Authors:** Jinguo Li, Fei Wang, Lili Song, Fang Fang, Mengru Shen, Hongyu Wang

**Affiliations:** 1 Graduate School, Bengbu Medical University, Bengbu, China; 2 Nursing Department, The Third People's Hospital of Bengbu, Bengbu, China; 3 School of Humanities and Health, Bengbu Medical University, Bengbu, China

**Keywords:** aging, Sarcopenia, maintenance hemodialysis, stage-based assessment, physical activity, nutritional status, self-efficacy, public health intervention

## Abstract

**Objectives:**

To investigate the stage-specific prevalence and progression mechanisms of sarcopenia in aging patients undergoing maintenance hemodialysis (MHD), and to identify modifiable risk and protective factors relevant to early public health interventions.

**Methods:**

This multicenter cross-sectional study enrolled 448 eligible older adults (aged ≧ 60 years) undergoing maintenance hemodialysis (MHD) from three tertiary hospitals in Bengbu, China, between January and April 2025, using convenience sampling. Sarcopenia was classified according to the Asian Working Group for Sarcopenia (AWGS) 2019 criteria. Data on demographics, body composition, nutrition, self-efficacy, physical activity, and inflammation were collected. Logistic regression analyses identified factors associated with sarcopenia onset and progression.

**Results:**

Sarcopenia prevalence was 54.0%, with 25.0% at the possible stage, 19.6% confirmed, and 9.4% severe. Key protective factors for sarcopenia onset included female sex, higher basal metabolic rate (BMR), higher Body Mass Index (BMI) and greater self-efficacy (SES6). Risk factors included low physical activity, diabetes, longer dialysis vintage, and malnutrition (MQSGA). Progression to confirmed/severe stages was independently associated with reduced BMI, protein mass, and self-efficacy, along with elevated BMR and physical inactivity. These findings highlight the importance of early screening and personalized preventive strategies in aging MHD populations.

**Conclusion:**

Sarcopenia is highly prevalent among older adults receiving MHD, with distinct stage-specific progression patterns. This study identified key modifiable risk and protective factors related to both the onset and progression of sarcopenia. Early detection of possible sarcopenia and timely interventions targeting nutrition, physical activity, and self-efficacy may delay progression and promote healthy aging in this population.

## 1 Introduction

With global population aging accelerating, sarcopenia has emerged as a major public health concern among older adults. Characterized by progressive loss of skeletal muscle mass, strength, and physical performance, sarcopenia is strongly associated with functional decline, increased risk of falls and fractures, hospitalization, and mortality. It imposes a significant burden on healthcare systems and threatens the successful implementation of healthy aging strategies (1).

Among older populations with chronic diseases, patients receiving MHD are at particularly high risk for developing sarcopenia (2, 3). The pathogenesis in this population is multifactorial and includes protein-energy wasting (PEW), chronic inflammation, dialysis-related metabolic disturbances, reduced physical activity, and psychosocial stressors (4). These mechanisms lead to accelerated muscle loss and functional impairment, contributing to poor quality of life and adverse clinical outcomes. As such, MHD patients represent a key target group for sarcopenia prevention and early intervention strategies in aging-related public health planning (5).

To address the need for early detection, the Asian Working Group for Sarcopenia (AWGS) introduced the concept of “possible sarcopenia” in its 2019 consensus, referring to a stage where muscle strength and/or physical function are reduced, but muscle mass remains within normal limits (6). This stage is considered potentially reversible and critical for preventive interventions (7, 8). However, current evidence on the epidemiological characteristics, clinical significance, and progression mechanisms of “possible sarcopenia” in the MHD population remains limited.

To fill this gap, the present study employed the AWGS 2019 staging criteria to assess the prevalence and stage-specific features of sarcopenia in a multicenter sample of MHD patients. We aimed to identify modifiable risk and protective factors associated with both the onset and progression of sarcopenia, thereby providing a scientific basis for early screening, stratified intervention, and public health decision-making in older hemodialysis patients. This evidence is expected to support healthy aging goals and improve outcomes in this vulnerable population.

## 2 Materials and methods

### 2.1 Study design and participants

This study is a multi-center cross-sectional study conducted with convenience sampling. The participants were selected from patients receiving MHD treatment at blood purification centers in three Grade A tertiary hospitals in Bengbu city from January to April 2025. The inclusion criteria were: (1) aged 60 years or older; (2) receiving hemodialysis for at least 3 months; (3) undergoing dialysis 2–3 times per week, with each session lasting 4 h and a blood flow rate of no less than 200 mL/min; (4) able to communicate and complete questionnaires and physical examinations; (5) voluntarily participating in the study and providing informed consent. The exclusion criteria included: (1) severe cognitive impairment or mental illness that prevented cooperation; (2) acute severe diseases within the past 6 months, such as infections, malignant tumors, or decompensated heart failure; (3) physical disabilities or metal implants (e.g., pacemaker) that precluded bioelectrical impedance testing; (4) use of glucocorticoids or immunosuppressants within the last 6 months; (5) pregnancy or lactation. This study was approved by the Ethics Committee of Bengbu Medical University (Ethical Approval Number: [2025] No. 187), and conducted in accordance with the principles of the Declaration of Helsinki. All participants provided written informed consent.

### 2.2 Survey tools

#### 2.2.1 General information questionnaire

This questionnaire, designed by the research team based on literature review and clinical expertise, was used to collect the basic demographic and disease-related information of the patients. It includes: (1) demographic data: gender, age, smoking and drinking habits, etc.; (2) disease characteristics: dialysis vintage, comorbid diabetes, etc.; (3) physical measurements: height, weight, grip strength, BMI, and pre-dialysis blood pressure; (4) body composition parameters: protein content, BMR measured by bioelectrical impedance analysis (BIA); and (5) laboratory indicators: C-reactive protein (CRP), serum albumin, hemoglobin, serum creatinine, calcium, phosphate, and Kt/V.

#### 2.2.2 Modified quantitative subjective global assessment (MQSGA)

The MQSGA, developed by Kalantar-Zadeh et al. (9), was used to assess the nutritional status of patients. It includes 7 items: weight changes, dietary intake, gastrointestinal discomfort, daily functioning, complications, subcutaneous fat loss, and muscle wasting. Each item is rated on a scale from 1 to 5, with a total score ranging from 7 to 35. Higher scores indicate greater nutritional risk or malnutrition.

#### 2.2.3 International physical activity questionnaire - short form (IPAQ-SF)

Physical activity was assessed using the Chinese version of the IPAQ-SF (10), which includes 7 items related to vigorous-intensity, moderate-intensity, walking, and sitting activities performed during the past week. Activity energy expenditure is calculated using metabolic equivalents (MET): vigorous activity = 8.0 MET, moderate activity = 4.0 MET, walking = 3.3 MET. Based on the IPAQ guidelines, physical activity is classified into low, moderate, and high levels according to the total MET value and activity type (11).

#### 2.2.4 Self-efficacy for managing chronic disease 6-item scale (SES6)

The SES6, developed by Stanford University, assesses patients’ self-efficacy in managing chronic diseases (12). It includes 6 items related to confidence in controlling symptoms, emotional regulation, maintaining physical function, and performing daily activities. Each item is rated from 0 to 10, with higher scores indicating stronger health behavior execution and self-regulation. The average score of all items reflects the patient’s overall self-efficacy (13).

#### 2.2.5 Sarcopenia diagnosis criteria

Sarcopenia screening and diagnosis in this study were based on the AWGS 2019 (14) guidelines, considering muscle mass, strength, and physical function. Muscle strength was measured using a handgrip dynamometer, with values <28 kg for men and <18 kg for women indicating muscle strength decline. Physical function was assessed using the 6-meter walking test, with speeds <1.0 m/s indicating functional decline. Muscle mass was measured by BIA to calculate the appendicular skeletal muscle index (ASM/height^2^), with <7.0 kg/m^2^ for men and <5.7 kg/m^2^ for women indicating low muscle mass. Sarcopenia is categorized into three stages: Possible Sarcopenia, where there is a decline in muscle strength and/or physical function but muscle mass is not yet reduced; Confirmed Sarcopenia, where low skeletal muscle mass is combined with either muscle strength decline or physical function reduction; and Severe Sarcopenia, which involves low skeletal muscle mass, muscle strength decline, and physical function reduction.

#### 2.2.6 Body composition and basal metabolic rate assessment

Body composition was assessed using the BIA method. Measurements were performed using the InBody 770 multi-frequency bioelectrical impedance analyzer (manufactured by Biospace, Korea). All measurements were conducted with the patient in a standing position. To minimize the influence of fluid shifts related to dialysis therapy, all body composition measurements were scheduled and performed within 30 min to 2 h following the patient’s mid-week dialysis session. Before measurement, participants were instructed to empty their bladder and bowels and to remove socks and metal objects. The assessment was carried out by uniformly trained research personnel following the device’s standard operating procedures.

The InBody 770 reports protein mass and calculates BMR using proprietary algorithms that incorporate measured fat-free mass and other impedance derived parameters. In this study, protein mass (kg) was used as a proxy for whole body protein reserves and skeletal muscle protein stores.

### 2.3 Data collection and quality control

To ensure data consistency and comparability and to minimize the potential influence of the dialysis treatment itself on patients’ subjective feelings and physiological state, all questionnaire assessments (including MQSGA, IPAQ-SF, and SES6) as well as grip strength and gait speed tests were uniformly conducted before the initiation of their mid-week dialysis session, when their physical condition was relatively stable.

Data were collected concurrently across all participating dialysis centers by research personnel who received standardized training. Prior to study initiation, all investigators were instructed on questionnaire administration, measurement techniques, and device operation to ensure procedural consistency and data reliability. Eligible participants were informed in detail about the study’s purpose and procedures and provided written informed consent before enrollment. Demographic and clinical information, as well as subjective assessments (MQSGA, IPAQ-SF, SES6), were obtained through structured face-to-face interviews. All forms were reviewed by a second investigator to ensure completeness and logical consistency.

Handgrip strength was measured prior to dialysis using an electronic dynamometer. Participants stood upright and held the device with their non-fistula hand, arm naturally extended. The test was performed three times, and the highest value was used in analysis. Gait speed was evaluated using a 6-meter walking test, with the mean value of two trials at a comfortable walking pace recorded. Laboratory data, including CRP, hemoglobin, serum albumin, creatinine, calcium, and phosphate, were extracted from pre-dialysis venous blood and analyzed in the hospital’s certified laboratory. All data were anonymized using coded identifiers and managed by designated personnel, with regular audits performed to ensure data integrity and traceability.

### 2.4 Statistical analysis

All data analyses were performed using SPSS version 26.0 (IBM Corp., Armonk, NY, USA). The distribution of continuous variables was assessed for normality. Variables with a normal distribution were presented as mean ± standard deviation (SD) and compared using independent-sample t-tests. Non-normally distributed variables were reported as median with interquartile range (IQR) and compared using the Mann–Whitney U test. Categorical variables were summarized as frequencies and percentages, and intergroup comparisons were conducted using the chi-square (χ^2^) test. For ordinal variables such as physical activity level, the Kruskal–Wallis H test was applied to assess differences across groups.

To identify factors associated with both sarcopenia occurrence and progression, two separate logistic regression analyses were conducted. The dependent variables were defined as (1) presence of sarcopenia (yes vs. no) and (2) sarcopenia severity (confirmed/severe vs. possible). Variables with a *p* value < 0.05 in univariate analyses were included in multivariate logistic regression models. For each model, regression coefficients (*β*), standard errors, Wald χ^2^ values, *p* values, odds ratios (OR), and 95% confidence intervals (95% CI) were reported. Statistical significance was set at a two-sided *p* < 0.05.

## 3 Results

### 3.1 Prevalence and classification of sarcopenia in MHD patients

A total of 448 MHD patients were included in this study. Sarcopenia was classified according to the criteria established by the AWGS 2019. Among them, 206 patients (46.0%) were classified as without sarcopenia, 112 (25.0%) as possible sarcopenia, and 130 (29.0%) as confirmed sarcopenia. Of the confirmed cases, 42 (9.4% of all participants and 32.3% of those with confirmed sarcopenia) were further identified as having severe sarcopenia. The detailed distribution of sarcopenia classifications is shown in Table 1.

**Table 1:** Prevalence and classification of sarcopenia in MHD patients (*n* = 448).

Classification of sarcopenia	Number of patients (*n*)	Proportion (%)
Without sarcopenia	206	46.0%
Possible sarcopenia	112	25.0%
Confirmed sarcopenia (including severe)	130	29.0%
of which: severe sarcopenia	42	9.4%
Total	448	100.0%

### 3.2 Clinical characteristics comparison between patients with and without sarcopenia

In this study, patients were classified into those with sarcopenia (*n* = 242), which included those with possible sarcopenia and confirmed sarcopenia (including severe cases), and those without sarcopenia (*n* = 206). The comparison of clinical characteristics between the two groups is shown in Table 2. Patients with sarcopenia had a significantly higher proportion of females (47.1% vs. 34.0%, *p* = 0.005), a longer median dialysis vintage [55.5 (26.0, 83.0) months vs. 35.0 (16.75, 56.0) months, *p* < 0.001], and a lower BMR [1,294 kcal/d vs. 1,503 kcal/d, *p* < 0.001]. The prevalence of comorbid diabetes was higher among those with sarcopenia (55.4% vs. 44.7%, *p* = 0.024). Regarding body composition and functional parameters, patients with sarcopenia had significantly lower BMI, protein mass, handgrip strength, and SES6 scores compared to those without sarcopenia (*p* < 0.001 for all). The MQSGA score was significantly higher in patients with sarcopenia (13 vs. 8, *p* < 0.001), indicating poorer nutritional status. Patients with sarcopenia also exhibited significantly lower physical activity levels, with 69.4% classified as having low physical activity, compared to only 8.7% in those without sarcopenia (*p* < 0.001). Additionally, CRP levels were significantly higher in patients with sarcopenia (*p* < 0.001), suggesting increased inflammation.

**Table 2:** Characteristics of the patients.

Variables		Possible or confirmed sarcopenia	Without sarcopenia	*p*
		(*n* = 242)	(*n* = 206)	
Gender [*n* (%)]	Male	128 (52.9)	137 (66)	
	Female	114 (47.1)	70 (34)	0.005
Age [M (*P*_25,_ *P*_75_), years]		67 (62, 74)	66 (61, 73)	0.134
Smoking status [*n* (%)]	Never	154 (63.6)	118 (57.3)	
	Formerly	46 (19)	53 (25.7)	0.221
	Currently	42 (17.4)	35 (17.0)	
Drinking status [*n* (%)]	Never	135 (55.8)	109 (52.9)	
	Formerly	77 (31.8)	67 (32.5)	0.750
	Currently	30 (12.4)	30 (14.6)	
Dialysis vintage [M (*P*_25_, *P*_75_), months]		55.5 (26, 83)	35 (16.75, 56)	<0.001
BMR[M (*P*_25_, *P*_75_), kcal/d]		1294 (1171, 1429)	1503 (1363, 1686)	<0.001
Comorbid diabetes [*n* (%)]	Yes	134 (55.4)	92 (44.7)	0.024
	No	108 (44.6)	114 (55.3)	
MQSGA Score [M (*P*_25_, *P*_75_), points]		13 (11, 14)	8 (8, 10)	<0.001
Physical activity level [*n* (%)]	Low	168 (69.4)	18 (8.7)	<0.001
	Moderate	66 (27.3)	74 (35.9)	
	High	8 (3.3)	114 (55.3)	
BMI[*M* (*P*_25_, *P*_75_), kg/m^2^]		22 (19.85, 24.35)	24.9 (23, 27.5)	<0.001
Protein [M (*P*_25_, *P*_75_), kg]		8.3 (7.1, 9.5)	10.45 (8.9, 11.8)	<0.001
SES6 Score[*M* (*P*_25_, *P*_75_), points]		4.7 (3.575, 5.85)	8.5 (7.8, 9)	<0.001
Handgrip Strength[*M* (*P*_25_, *P*_75_), kg]		15.3 (11.625, 17.75)	29.3 (22.3, 34.0)	<0.001
CRP[*M* (*P*_25_, *P*_75_), mg/L]		3.66(2.29, 5.688)	2.5 (0.77, 3.8)	<0.001
Serum Albumin[*M* (*P*_25_, *P*_75_), g/L]		38.77 (36.69, 41.54)	39.91 (38.07, 41.68)	0.042
Hemoglobin (g/L)		98.62 ± 15.49	97.09 ± 16.83	0.601
Creatinine [M (*P*_25_, *P*_75_), μmol/L]		891.00 (767.00, 1088.00)	996.00 (763.00, 1122.00)	0.259
Calcium (mmol/L)		2.22 ± 0.21	2.18 ± 0.21	0.483
Phosphate [M (*P*_25_, *P*_75_), mmol/L]		2.08 (1.68, 2.33)	2.30 (1.92, 2.65)	0.016
Kt/V [M (*P*_25_, *P*_75_)]		1.45(1.34, 1.69)	1.28 (1.16, 1.41)	<0.001
Systolic BP (mmHg)		122.52 ± 14.40	132.16 ± 15.47	<0.001
Diastolic BP (mmHg)		74.32 ± 10.44	76.26 ± 12.43	0.07

BMI: Body Mass Index; MQSGA Score: Modified Quantitative Subjective Global Assessment score; SES6 Score: Self-Efficacy for Managing Chronic Disease 6-item scale score; BMR: Basal Metabolic Rate; CRP: C-Reactive Protein; Kt/V: Dialysis adequacy index.

### 3.3 Analysis of factors influencing possible or confirmed sarcopenia in MHD patients

Logistic regression analysis was performed using patients without sarcopenia as the control and those with possible or confirmed sarcopenia as the case group. In the multivariable analysis, female sex (OR = 0.023, 95% CI: 0.001–0.402, *p* = 0.010), higher self-efficacy (SES6 score) (OR = 0.129, 95% CI: 0.062–0.269, *p* < 0.001), higher BMR (OR = 0.987, 95% CI: 0.976–0.998, *p* = 0.023), and higher BMI (OR = 0.738, 95% CI: 0.565–0.962, *p* = 0.025) were identified as protective factors against sarcopenia. Comorbid diabetes (OR = 4.639, 95% CI: 1.061–20.275, *p* = 0.041), longer dialysis vintage (OR = 1.025, 95% CI: 1.005–1.045, *p* = 0.013), poorer nutritional status (MQSGA score) (OR = 2.778, 95% CI: 1.710–5.450, *p* < 0.001), and low physical activity level (IPAQ: low vs. high: OR = 26.307, 95% CI: 2.802–247.004, *p* = 0.004) were identified as significant independent risk factors for sarcopenia (Table 3).

**Table 3:** Univariable and multivariable logistic regression analysis: factors affecting the possible or confirmed sarcopenia (*n* = 448).

Variables	Univariable analysis			Multivariable analysis		
	OR	95% CI	*p*	OR	95% CI	*p*
Gender						
Male	Reference			Reference		
Female	1.730	1.180–2.538	0.005	0.023	0.001–0.402	0.010
Age	1.013	0.998–1.028	0.089			
Smoking status						
Never	Reference					
Formerly	0.891	0.569–1.394	0.612			
Currently	1.108	0.670–1.832	0.690			
Drinking Status						
Never	Reference					
Formerly	0.928	0.614–1.403	0.723			
Currently	0.807	0.459–1.421	0.458			
Dialysis vintage	1.014	1.009–1.020	<0.001	1.025	1.005–1.045	0.013
BMR	0.995	0.993–0.996	<0.001	0.987	0.976–0.998	0.023
Comorbid diabetes						
Yes	1.537	1.058–2.234	0.024	4.639	1.061–20.275	0.041
No	Reference			Reference		
MQSGA Score	2.846	2.358–3.436	<0.001	2.778	1.71–5.450	<0.001
Physical activity level						
Low	133.000	55.937–316.229	<0.001	26.307	2.802–247.004	0.004
Moderate	12.709	5.769–28.002	<0.001	7.031	0.851–58.092	0.07
High	Reference			Reference		
BMI	0.758	0.70–90.810	<0.001	0.738	0.565–0.962	0.025
Protein	0.536	0.471–0.611	<0.001	0.983	0.269–3.584	0.979
SES6 Score	0.109	0.069–0.172	<0.001	0.129	0.062–0.269	<0.001
CRP	1.046	1.013–1.080	0.007	1.086	0.969–1.216	0.156

BMI: Body Mass Index; MQSGA Score: Modified Quantitative Subjective Global Assessment score; SES6 Score: Self-Efficacy for Managing Chronic Disease 6-item scale score; BMR: Basal Metabolic Rate; CRP: C-Reactive Protein.

### 3.4 Multivariate logistic regression analysis of factors influencing sarcopenia progression

To further identify the key factors influencing the progression of sarcopenia from the “possible” stage to the “confirmed or severe” stages, this study classified patients with confirmed or severe sarcopenia as the case group (*n* = 130), while possible sarcopenia patients were the control group (*n* = 112). Multivariate logistic regression analysis was conducted, as detailed in Table 4. Factors such as physical activity levels, body composition, metabolic indicators, and self-efficacy were all closely related to sarcopenia progression. Low physical activity levels significantly increased the risk of sarcopenia progression, with a risk increase of over 50 times compared to high physical activity levels (OR = 54.722, 95% CI: 2.224–962.841, *p* = 0.007). A decrease of 1 kg/m^2^ in BMI increased the risk of progression by approximately 51% (OR = 0.492, 95% CI: 0.378–0.646, *p* < 0.001). For every increase of 100 kcal/d in BMR, the risk increased by about 4.8% (OR = 1.048, 95% CI: 1.004–1.092, *p* = 0.042). A decrease of 1 kg in protein content significantly increased the risk of progression (OR = 0.004, 95% CI: 0.001–0.343, *p* = 0.020). For every 1-point decrease in SES6 score, the risk of sarcopenia progression increased by about 44% (OR = 0.558, 95% CI: 0.344–0.903, *p* = 0.018).

**Table 4:** Univariable and multivariable logistic regression analysis: confirmed sarcopenia + severe sarcopenia vs. possible sarcopenia (*n* = 242).

Variables	Univariable analysis			Multivariable analysis		
	OR	95% CI	*p*	OR	95% CI	*p*
Gender						
Male	Reference					
Female	1.202	0.724–1.996	0.476			
Age	1.015	0.993–1.038	0.181			
Smoking status						
Never	Reference					
Formerly	0.784	0.421–1.459	0.442			
Currently	1.197	0.610–2.349	0.602			
Drinking status						
Never	Reference					
Formerly	0.797	0.454–1.397	0.428			
Currently	0.776	0.351–1.715	0.531			
Dialysis vintage	1.014	1.009–1.020	<0.001	1.012	0.998–1.024	0.129
BMR	0.995	0.993–0.996	<0.001	1.048	1.004–1.092	0.042
Comorbid diabetes						
Yes	1.001	0.602–1.664	0.997			
No	Reference					
MQSGA Score	2.846	2.358–3.436	<0.001	0.824	0.726–1.173	0.574
Physical activity level						
Low	133.000	55.937–316.229	<0.001	54.722	2.224–962.841	0.007
Moderate	12.709	5.769–28.002	<0.001	22.351	1.245–283.964	0.034
High	Reference			Reference		
BMI	0.758	0.709–0.810	<0.001	0.492	0.378–0.646	<0.001
Protein	0.536	0.471–0.611	<0.001	0.004	0.001–0.343	0.020
SES6 Score	0.109	0.069–0.172	<0.001	0.558	0.344–0.903	0.018
CRP	1.046	1.013–1.080	0.007	1.077	0.992–1.171	0.072

BMI: Body Mass Index; MQSGA Score: Modified Quantitative Subjective Global Assessment score; SES6 Score: Self-Efficacy for Managing Chronic Disease 6-item scale score; BMR: Basal Metabolic Rate; CRP: C-Reactive Protein.

## 4 Discussion

In older MHD patients aged 60 years or above, the prevalence of possible or confirmed sarcopenia is relatively high (54%). Sarcopenia in MHD patients exhibits a graded progression from “possible” to “confirmed” and “severe,” with proportions of 25.0, 19.6, and 9.4%, respectively. This study revealed differences in body composition, physical activity levels, nutritional status, and self-management abilities across different stages of sarcopenia in MHD patients, highlighting the importance of early identification and intervention. In the “possible sarcopenia” stage, although muscle mass has not yet significantly declined, there is already noticeable deterioration in physical function or muscle strength, making it a critical period for delaying disease progression (15). Patients in this stage account for nearly one-quarter of the population, and early intervention at this point could potentially prevent progression to confirmed or severe sarcopenia (16). Severe sarcopenia accounts for more than one-third of the confirmed sarcopenia cases, indicating that some patients are already in a severe stage at the time of the first assessment, reflecting the current issue of “delayed recognition” in clinical practice.

This study identified several key, modifiable factors, specifically nutritional status, physical activity, and self-efficacy, that are independently associated with sarcopenia in aging MHD patients. Malnutrition was identified as a major determinant of sarcopenia in MHD patients (17). MQSGA comprehensively assesses dietary intake, gastrointestinal symptoms, and signs of muscle or fat loss (9), while serum albumin provides an objective biochemical marker of protein-energy status. In this study, patients with sarcopenia showed significantly higher MQSGA scores and lower serum albumin levels than those without sarcopenia. The findings suggest that deteriorating nutrition contributes to sarcopenia through insufficient amino acid availability, anabolic resistance, and persistent inflammation, which accelerate muscle protein breakdown (18). Although serum albumin may be influenced by hydration or inflammation, its combination with MQSGA offers a more comprehensive assessment of nutritional risk.

Inadequate physical activity represents another crucial factor. IPAQ results suggest that physical activity is seriously insufficient among dialysis patients. In this study, low levels of physical activity appear to be not only a potential contributor to the onset of sarcopenia but also a significant risk factor for its progression from the “possible” to the “confirmed/severe” stages. This aligns with the recognized decline in physical capacity and musculoskeletal function associated with sarcopenia, especially in its severe form (6). Low levels of physical activity mean that skeletal muscles are chronically deprived of effective loading, which may reduce the recruitment of type II muscle fibers and lead to decreased muscle tone and responsiveness (19–22). Among patients receiving MHD, common symptoms such as dialysis-related fatigue, myalgia, and joint stiffness can further exacerbate the vicious cycle of reduced mobility and progressive muscle atrophy (23–25). However, given the cross-sectional design of this study, it is not possible to establish a causal link between low physical activity and sarcopenia progression. Longitudinal research is warranted to explore this relationship further.

The SES6 score provides a validated measure of patients’ self-efficacy in managing their disease and engaging in health-promoting behaviors, including their confidence and capability in maintaining exercise routines, dietary regulation, and daily activity management (13). In this study, lower SES6 scores were significantly associated with an increased risk of both the onset and progression of sarcopenia. Evidence suggests that patients with lower levels of self-efficacy often lack the motivation and capacity to sustain healthy behaviors, such as regular exercise, adequate nutrition, and participation in rehabilitation, which may in turn accelerate the decline in muscle mass and function (26). Among MHD patients, dialysis-related fatigue, psychological distress, and diminished quality of life can further undermine self-management capacity, potentially weakening the engagement in muscle-preserving behaviors (27). Previous studies have demonstrated that enhancing self-efficacy can improve treatment adherence and overall quality of life (28).

During the progression phase of sarcopenia, reduced protein mass emerges as an independent risk factor. The underlying mechanism involves several key aspects: total body protein primarily reflects protein content in skeletal muscle, internal organs, and other tissues, with skeletal muscle protein constituting the predominant component. Therefore, a decline in total protein directly indicates reduced skeletal muscle mass and diminished muscle protein reserves (29). In patients undergoing MHD, this process is further exacerbated by chronic inflammation, metabolic disturbances, and sustained protein losses, which collectively suppress muscle protein synthesis while accelerating protein degradation, ultimately leading to gradual depletion of protein mass (30). Compared to serum markers such as albumin, protein mass measured by BIA devices offers distinct advantages in clinical practice, as it can more conveniently reflect dynamic changes in muscle tissue, particularly when body weight remains relatively stable, thereby providing valuable complementary information for monitoring alterations in muscle reserves.

The findings collectively demonstrate significant associations between sarcopenia in MHD patients and nutritional, behavioral, and psychosocial factors. MQSGA, IPAQ-SF, and SES6 reflect these distinct dimensions, together providing a multidimensional understanding of sarcopenia risk. Although the cross-sectional design precludes causal inferences, these findings offer valuable insights: incorporating these assessment tools into routine clinical monitoring may facilitate early identification and risk stratification of high-risk populations.

Building on the observed associations, we propose a staged management approach: at the preliminary stages, emphasis could be placed on nutritional support, physical activity promotion, and self-efficacy building; for patients already in the “possible” stage, particular attention should be paid to maintaining protein reserves and addressing physical inactivity. The effectiveness of these management approaches requires validation through prospective studies. Future research should include longitudinal studies to establish causal relationships between these factors and disease progression, and subsequently develop and test targeted intervention strategies based on these findings.

This study found that the BMR plays a complex role at different stages of sarcopenia in MHD patients. At the onset stage of sarcopenia, a higher BMR was associated with a lower risk of sarcopenia, suggesting that a favorable energy metabolism state may help maintain muscle mass, potentially by promoting protein synthesis and inhibiting protein degradation. This finding is consistent with previous studies (31). However, as sarcopenia progresses, an increased BMR becomes a risk factor. Existing literature suggests that dialysis patients often experience chronic low-grade inflammation (elevated IL-6 and TNF-*α*), which directly stimulates basal metabolism, leading to increased resting energy expenditure and an abnormally elevated BMR. Simultaneously, protein synthesis is inhibited, and muscle protein degradation is accelerated, resulting in a state of metabolic inefficiency (32–34). An elevated BMR at this stage likely reflects a pathological process of intensified energy consumption and muscle loss, rather than a simple increase in metabolic activity (35). The observed relationship between BMR and sarcopenia may also be confounded by inflammation, body composition, and sex-specific hormonal differences (36). Inflammation can blur this association because it not only elevates metabolic rate but also coexists with malnutrition and muscle wasting, making it difficult to distinguish whether high BMR represents adaptive metabolism or disease burden. Body composition is another major confounder, as BMR is predominantly determined by fat-free mass, particularly skeletal muscle, which has a much higher metabolic rate than adipose tissue. Therefore, individuals with a greater proportion of adipose tissue may exhibit a similar absolute BMR but lower metabolic efficiency per unit of lean mass. In addition, fluid retention and intramuscular fat infiltration in MHD patients may distort the estimation of lean tissue, leading to potential bias in BMR interpretation. Sex-specific hormonal differences may further modify this relationship: lower testosterone levels in men and estrogen-driven metabolic regulation in women can differentially influence substrate utilization and energy efficiency (37, 38). These overlapping influences suggest that BMR should not be interpreted in isolation but rather within an integrated physiological framework that accounts for inflammation, body composition, and sex-related characteristics. Future longitudinal studies using indirect calorimetry, precise body composition measurements, and inflammatory markers are warranted to validate and elucidate the causal and temporal relationships between BMR alterations and sarcopenia progression in MHD populations.

This study has several limitations. First, its cross-sectional design precludes causal inference, and longitudinal studies are needed to clarify temporal relationships and verify predictive value. Second, the study sample was drawn from three hospitals in Bengbu, China, which may restrict the external validity and generalizability of the findings. Third, some measures (such as BMR) were derived from bioimpedance-based estimates that can be influenced by hydration status and device-specific algorithms (39). Fourth, both IPAQ-SF and SES6 were self-reported instruments and may be subject to recall and social desirability bias. Future multicenter, prospective studies with more diverse populations and standardized multidimensional assessments are warranted to validate these findings and provide stronger evidence for clinical guidelines.

## 5 Conclusion

This study systematically investigated the prevalence and stage-specific risk factors of sarcopenia in older adults receiving MHD using the AWGS 2019 criteria. A high prevalence of sarcopenia was observed, with a clear pattern of stage-wise progression. During the onset phase, protective factors included female sex, higher self-efficacy, and elevated BMR, whereas low physical activity, diabetes, extended dialysis duration, and malnutrition were major risk contributors. Independent predictors for progression from possible to confirmed sarcopenia included reduced physical activity, declining BMI, decreased protein mass, and diminished self-efficacy. Interestingly, BMR demonstrated bidirectional changes across different stages, implying a complex regulatory role in sarcopenia development. These findings underscore the importance of early recognition of possible sarcopenia, regular monitoring of body composition and metabolic parameters, and implementing integrated interventions focusing on nutrition, physical activity, and self-management. Such strategies may help delay sarcopenia onset and progression, ultimately improving long-term outcomes. Future large-scale, multicenter, longitudinal studies are warranted to further elucidate the underlying mechanisms and validate intervention strategies.

## Data Availability

The raw data supporting the conclusions of this article will be made available by the authors, without undue reservation.
